# Evidence of association between Nucleosome Occupancy and the Evolution of Transcription Factor Binding Sites in Yeast

**DOI:** 10.1186/1471-2148-11-150

**Published:** 2011-05-31

**Authors:** Krishna BS Swamy, Wen-Yi Chu, Chun-Yi Wang, Huai-Kuang Tsai, Daryi Wang

**Affiliations:** 1 Institute of Information Science, Academia Sinica, Taipei, 115, Taiwan; 2 Bioinformatics Program, Taiwan International Graduate Program, Academia Sinica, Taipei, 115, Taiwan; 3 Institute of Biomedical Informatics, National Yang-Ming University, Taiwan; 4 Department of Computer Science and Information Engineering, National Taiwan University, Taiwan; 5 Biodiversity Research Center, Academia Sinica, Taipei, 115, Taiwan; 6 Research Center for Information Technology Innovation, Academia Sinica, Taipei, 115, Taiwan

## Abstract

**Background:**

Divergence of transcription factor binding sites is considered to be an important source of regulatory evolution. The associations between transcription factor binding sites and phenotypic diversity have been investigated in many model organisms. However, the understanding of other factors that contribute to it is still limited. Recent studies have elucidated the effect of chromatin structure on molecular evolution of genomic DNA. Though the profound impact of nucleosome positions on gene regulation has been reported, their influence on transcriptional evolution is still less explored. With the availability of genome-wide nucleosome map in yeast species, it is thus desirable to investigate their impact on transcription factor binding site evolution. Here, we present a comprehensive analysis of the role of nucleosome positioning in the evolution of transcription factor binding sites.

**Results:**

We compared the transcription factor binding site frequency in nucleosome occupied regions and nucleosome depleted regions in promoters of old (orthologs among Saccharomycetaceae) and young (Saccharomyces specific) genes; and in duplicate gene pairs. We demonstrated that nucleosome occupied regions accommodate greater binding site variations than nucleosome depleted regions in young genes and in duplicate genes. This finding was confirmed by measuring the difference in evolutionary rates of binding sites in *sensu stricto *yeasts at nucleosome occupied regions and nucleosome depleted regions. The binding sites at nucleosome occupied regions exhibited a consistently higher evolution rate than those at nucleosome depleted regions, corroborating the difference in the selection constraints at the two regions. Finally, through site-directed mutagenesis experiment, we found that binding site gain or loss events at nucleosome depleted regions may cause more expression differences than those in nucleosome occupied regions.

**Conclusions:**

Our study indicates the existence of different selection constraint on binding sites at nucleosome occupied regions than at the nucleosome depleted regions. We found that the binding sites have a different rate of evolution at nucleosome occupied and depleted regions. Finally, using transcription factor binding site-directed mutagenesis experiment, we confirmed the difference in the impact of binding site changes on expression at these regions. Thus, our work demonstrates the importance of composite analysis of chromatin and transcriptional evolution.

## Background

The chromatin of eukaryotic genomes is compacted into several levels. Nucleosomes, which form the lowest level of compaction, are made up of ~147 bp of DNA wrapped around a histone protein complex and interspersed by ~50 bp of exposed linker DNA. In recent years, the occupancy of nucleosome positions in yeasts has been investigated by using different approaches (such as tiling arrays and parallel sequencing), which employs micrococcal nuclease (MNase) digestion [1-3]. The results show that about 70-80% of the yeast genome is occupied by nucleosomes [4-6]. The intrinsic mechanisms that determine the nucleosome locations have long been of interest to researchers. Studies of budding yeast have discovered dinucleotides (AA/TT/AT) periodicity along nucleosome positioning sequences [7,8]; and that nucleosome depleted regions (NDRs) are characterized by positioned stretches of poly (dA:dT) tracts [9,10]. In addition, a number of patterns of nucleosome occupancy have been observed. For example, a ~140 bp NDR is often found upstream of the transcription start site flanked by -1 and +1 nucleosomes, with the +1 nucleosome located ~13 bp downstream from the transcription start site [11,12]. It has also been found that, near the 5' end of genes, a uniform 165 bp spacing of nucleosomes (18 bp linker) extends to as many as nine nucleosomes [5-8,13-15]. Importantly, many of these features are evolutionary conserved [7,16].

It is known that the transcription mechanism in eukaryotes functions at different levels, e.g. at the DNA sequence level, transcription factors interact with *cis*-regulatory sequences; and at the chromatin level, where the chromatin allows the chromosomal segments to switch between activated state and suppressed states of transcription [17,18]. The interplay of changes in nucleosome occupancy and transcriptional machinery at each level suggests a strong association between nucleosome positioning and transcription mechanism [19,20]. For example, TATA-less promoters, which are characterized by NDRs, are frequently linked to basal transcription. Conversely, the promoters of TATA-containing genes tend to be occupied by nucleosomes and are stress responsive [13,21,22]. Moreover, it has been demonstrated that nucleosomes could facilitate the recognition of transcription factor binding sites (TFBSs), and guide transcription factors to their target sites in a DNA sequence [22,23]. As an example, Maffey *et al. *[24] characterized the constraints imposed by well positioned nucleosomes on the interaction of androgen receptors with their binding sites, which are located in the proximal promoters of murine probasin genes. The above evidence confirms the importance of the association between nucleosome positioning and transcriptional regulation. Such evidence in turn raises the interesting issue of the role of nucleosomes in constraining evolutionary changes in TFBSs.

Recent studies have identified the evolutionary features related to nucleosome organization in yeasts [9,25]. For example, it has been found that nucleosome free linker regions have a lower evolution rate than nucleosome occupied regions (NRs) [9,25]. In an another study, a large-scale comparative genomic analysis of distantly related yeasts found that gene expression divergence is coupled with the evolution of DNA-encoded nucleosome organization [26]. Further, by analyzing the nucleosome position of two closely related yeast species, Tirosh *et al. *[27] indicated that the major contribution towards divergence of nucleosome positioning is through mutations in the local sequences (*cis*-effects). Moreover, the sequences that quantitatively affect nucleosome occupancy were found to evolve under compensatory dynamics while maintaining heterogeneous levels of AT content [28]. Considering the fact that significant fraction of regulatory variation can be attributed to changes in *cis*-regulatory elements [29-32], understanding the evolutionary process requires the investigation of all the factors that contribute to TFBS evolution [33]. With the availability of the whole genome nucleosome map in yeast species [34], it is thus desirable to extend existing studies on regulatory regions from an evolutionary perspective while considering the presence of chromatin structure. In this paper, we have attempted a more comprehensive analysis to demonstrate that nucleosome occupancy in yeast promoters plays an important role in the evolutionary changes in TFBSs.

To determine the evolutionary features of TFBSs constrained by nucleosome occupancy, we first investigated the distribution of TFBSs in NRs and NDRs that regulate 1) orthologous genes of *Saccharoymyces cerevisiae, Candida glabrata, and Kluyveromyces lactis *(Saccharomycetaceae); and 2) those that specifically regulate *S. cerevisiae *(Saccharomyces specific) genes, which represent young genes. We found that TFBS locations in orthologous genes are dominant in NDRs, but those in Saccharomyces specific genes appear more frequently in NRs. To further validate this evolutionary tendency, we investigated the distribution of TFBSs in NRs and NDRs in duplicate gene pairs of yeast that might have undergone relaxation of selection pressure. Since TFBS variations are due to difference in consensus sequences and nucleotide substitutions can promote diversification of regulatory elements [35,36], these interesting findings motivated us to estimate the evolution of TFBSs by position-specific evolution rates [37]. The evolution rates of TFBSs were found to be higher at NRs than their depleted counterparts (NDRs). Finally, the impact of TFBS changes on gene expression at NRs and NDRs were evaluated using site-directed mutagenesis of TFBS and real-time PCR analysis. Our findings on the evolutionary events in TFBSs suggest that 1) NRs can accommodate more changes that contribute to the variation in TFBSs, and 2) the selection constraints of NRs and NDRs are different. Future analyses of data across different biological conditions can reflect on the role of variations in TFBSs.

## Methods

### Collecting yeast TFBSs

**Figure 1:**
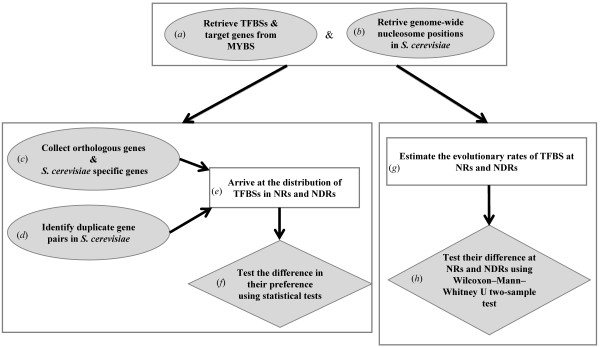
**Flowchart of the proposed method**. (*a*) The target genes and consensus of transcription factors in the three *sensu stricto *species (*S. cerevisiae*, *S. paradoxus *and *S. mikatae*) were downloaded from the MYBS database; (*b*) nucleosome positions in *S. cerevisiae *was compiled from Mavrich *et al. *[11]; (*c*) orthologous genes were collected from OrthoMCL-DB and detected *S. cerevisiae *specific genes; (*d*) duplicate gene pairs were identified in *S. cerevisiae*; (*e*) the frequency distribution of TFBSs in orthologous genes, sacharomyces specific genes and duplicate gene pairs were derived with respect to nucleosome occupancy in *S. cerevisiae*; (*f*) suitable statistical tests were used to determine if the distributions in (*e*) was significantly different; (*g*) the evolutionary rates of TFBS present in *sensu stricto *yeasts was calculated at NRs and NDRs; and (*h*) the difference in (*g*) were tested for significant difference.

The genome sequence and the gene and chromosome annotations of the yeast species examined in this study were obtained from a recent compilation in the Saccharomyces Genome Database (SGD) [38]. The target genes of transcription factors and their TFBSs in five closely related yeasts from the *Saccharomyces sensu stricto clade*, namely, *S. cerevisiae*, *S. paradoxus*, *S. mikatae*, *S. kudriavzevii *and *S. bayanus*, were retrieved from the MYBS database http://cg1.iis.sinica.edu.tw/~mybs/[39] (Figure 1a). MYBS contains integrated information derived from an array of experimentally verified and predicted consensus or position weight matrices (PWMs) that correspond to 183 known yeast transcription factors.

To improve the accuracy of binding site search, traditional methods impose filters such as phylogenetic footprinting information and transcription factor-DNA binding affinity by setting the *p*-value in a ChIP-chip experiment. However, during inter- or intra-species evolutionary analysis, using conservation of phylogenetic footprinting as primary criteria will not be feasible. In such cases, simply considering the constraints of bound promoters in ChIP-chip data might be insufficient. Thus, in this current work, to control for the specificity of TFBSs, we examined the reliable annotations of TFBSs for each transcription factor according to the following criterion. For a transcription factor α, the ratio

had to be satisfied. We applied an additional criterion that the *p*-value of the corresponding transcription factor ChIP-chip experiment for the gene should be ≤ 0.001 [40]. Furthermore, to avoid ambiguity, overlapping TFBSs corresponding to the same transcription factor were excluded from our analysis. In total, our dataset contained 104 transcription factors with 29,193 TFBSs in 2,522 promoters of *S. cerevisiae*. For TFBSs corresponding to the 104 transcription factors that occurred at least once in all five *sensu stricto *species, including *S. cerevisiae*, there were 22,447 TFBSs present in 1134 promoters (Table 1).

**Table 1:** Information about the target genes and the TFBSs studied

	***S. cerevisiae***^***a***^	***Sensu stricto *species**^***b***^	**Orthologous genes**^***c***^	**Saccharomyces specific genes**^***d***^
# of target genes	2522	1134	2152	75
# of TFBSs	29193	22447	23605	1144

***^a ^***Number of target genes and TFBSs of *S. cerevisiae *from our data set

***^b ^***Number of target genes and TFBSs that are present in promoters of all five *Sensu stricto yeasts*

***^c ^***Number of TFBSs in genes conserved in Saccharomycetaceae species (*S. cerevisiae*, *C. glabrata*, and *K. lactis*)

***^d ^***Number of TFBSs genes only present in *S. cerevisiae*

### Nucleosome occupancy information in *S. cerevisiae*

Genome-wide nucleosome occupancy data (Figure 1b) of *S. cerevisiae *was retrieved from http://atlas.bx.psu.edu/project/saccharomyces.html[11]. Mavrich *et al. *[11] used MNase digested DNA from nucleosome core particles that were crosslinked with formaldehyde *in vivo*. These were further immunopurified with antibodies against tagged histones H3 and H4. After correction for MNase bias and making calls on nucleosome locations, a total of 1,206,057 individual nucleosomal DNAs were sequenced using Roche GS20 (454 Life Sciences), and then mapped to genomic coordinates obtained from http://www.yeastgenome.org[38]. Furthermore, Mavrich *et al*. established rules governing genomic nucleosome organization in *S. cerevisiae*. They also developed a statistical model to predict nucleosome positions in terms of nucleosome occupancy, and identified well positioned and fuzzy nucleosomes. In this work, we consider both well positioned and fuzzy nucleosomes.

### Orthologous and Saccharomyces specific genes

We first examined the differential relationship between the frequency distribution of TFBSs in orthologous genes (in Saccharomycetaceae) and in genes only present in the descendent species *S. cerevisiae*, with respect to nucleosome occupancy. For this task, we collected the genome sequences of three diverged yeast species, namely, *S. cerevisiae*, *C. glabrata*, and *K. lactis*, from SGD [38]. Then, for each of the 2,522 genes in *S. cerevisiae*, we downloaded the "orthologous" genes in *C. glabrata *and *K. lactis *from the OrthoMCL-DB [41] (see Table 1 and Figure 1c). Genes that existed in *S. cerevisiae*, but not in *C. glabrata *or *K. lactis*, are called "Saccharomyces specific" genes. Additional file 1 Table S1 lists 2,152 orthologous genes and 75 Saccharomyces specific genes considered in our analysis.

Next, using the TFBSs from our set of transcription factors, we computed the numbers of TFBSs in the nucleosome occupied regions (NRs) and nucleosome depleted regions (NDRs) of each gene (Table 1) based on the genome wide nucleosome occupancy map of *S. cerevisiae *[11] (Figure 1e). The measurement was performed separately on the orthologous genes and Saccharomyces specific genes. A TFBS was deemed to be in NR (or NDR) if its location overlapped (or did not overlap) with that of the nucleosome positions retrieved from Mavrich *et al. *[11]. For those TFBSs, we used a two-sided *χ^2^*-test to determine whether the differences in their frequency in NRs and NDRs occurred more often than under random expectation (Figure 1f). The null hypothesis *H_0 _* is that the frequency distribution in NRs is equal to the distribution in NDRs, and the alternative hypothesis is that they are different. We rejected the null hypothesis under the criterion that the *p*-value ≤ 0.05.

### Identifying duplicate genes in *S. cerevisiae*

We compiled a list of 1,048 independent duplicate pairs in the *S. cerevisiae *genome by adopting a similar, but more stringent, protocol to that developed by Gu *et al. *[42]. First, we downloaded all available proteins in *S. cerevisiae *from the latest compilation of SGD [38]. To identify duplicate gene pairs (Figure 1d), we performed an all-against-all BLASTP search on the entire proteome. Two genes were regarded as duplicate pairs if they satisfied the following three criteria. First, the expected value (*E*) of reciprocal best hits during the BLASTP search should be < 10^-20^. Second, the length of the alignable region (*L*) between the two sequences should be greater than half of the length of the longer protein. Third, their similarity should be ≥ *I*, where *I *= 30% if *L *≥ 150 amino acids (a.a.); and *I *= 0.06 + 4.8*L*^-0.32(1 + exp(-L/1000)) ^if *L *< 150 a.a.. Furthermore, all overlapping pairs and transposons containing genes were excluded to ensure that each gene pair only occurred once in our dataset. Moreover, only gene pairs with at least 150 informative codons were retained for further analysis.

From the promoters of duplicate gene pairs, we computed the frequency of TFBSs in NRs and NDRs and normalized them with the total number of TFBSs at these regions (Figure 1e). Furthermore, we determined whether the preference of the TFBSs at NRs and NDRs were significantly different according to one-sided two-sample proportion test (Figure 1f) under the criterion that *p*-value < 0.01 (Table 2).

**Table 2:** The distribution of TFBSs in the NRs and NDRs of orthologous genes and Saccharomyces specific genes, and the distribution of TFBSs in duplicate gene pairs.

	# of TFBSs in NRs	# of TFBSs in NDRs
Orthologous genes	11710 (3.33)	11895 (3.67)
Saccharomyces specific genes ***χ^2 ^*= 9.0, *p*-value < 0.002**	620 (5.65)	524 (2.84)
Duplicate pairs **Z-value = 32.07, *p-value *= 1.25 × 10^-40^**	8793 (5.50)	4892 (3.06)

The values in parenthesis correspond to the number of TFBSs per gene. *χ*^*2*^-test *p*-value for ortholog and Saccharomyces specific gene sets and one-sided two sample proportion test results for duplicate genes pairs are also shown.

### Calculating the evolution rates of TFBSs

We calculated the evolution rates of TFBSs in NRs and NDRs based on the method proposed by Moses *et al. *[37]. The rates were computed for all the TFBSs of *S. cerevisiae *that were conserved in other *sensu stricto *yeasts (Figure 1g). Using aligned promoters from the same gene sets of *sensu stricto *yeasts [43], species tree of these species [44,45] and parsimony algorithm [46], we derived evolutionary inference by computing the minimal number of changes (minimum parsimony) needed to align each column of the promoters in all four species with the promoters of *S. cerevisiae*. Promoter regions with missing sequences in the alignment were treated as gaps and excluded.

The average evolution rate of a TFBS was obtained by computing the sum of the minimal number of changes over all positions, and then divided by its length. Given that mutation rates at NRs are higher across the genome when compared to NDRs [9,25], it could be intriguing whether the evolutionary rates of TFBSs at NRs and NDRs is an extrapolation of the genome-wide trend. In this situation, using the complete set of promoter sequences containing the TFBSs as a general background can induce considerable bias. Hence, to control for such context-inducible bias, we calculated the number of changes in NRs and NDRs separately, by excluding the positions containing TFBSs on each promoter. These two calculations act as two types of backgrounds. The number of changes in a TFBS (at NRs and NDRs) was further normalized by the number of changes in the respective background (Figure 1g). Furthermore, for species with short evolutionary distances, like those considered here, the number of substitutions per site of a DNA sequence determined by using parsimony methods is expected to be similar to that obtained by applying maximum likelihood approach. We also investigated whether the median evolution rate of TFBSs at NRs was statistically greater than that of TFBSs at NDRs by applying the Wilcoxon-Mann-Whitney U two-sample test with a stringent criterion that the *p*-value ≤ 0.01 (Figure 1h).

### Mutagenesis for TFBSs

A yeast strain (BY4741 (BY), a descendant of S288C) was grown in yeast extract-peptone-adenine-dextrose (YPAD) medium [47] and harvested at the mid-log phase. Overnight yeast cultures were used to prepare the starting cultures with OD_600 _= 0.1 and grown in the YPAD medium at 30°C with 250 rpm shaking. The yeast cells were harvested at the OD_600 _= 1.0, and the total RNAs were extracted by using the MasterPure™ yeast RNA purification kit (EPICENTRE), and contaminated DNAs were removed by treatment of DNaseI in the same kit.

To determine the effects of TFBSs that have recently evolved in related yeast species on expression difference, we randomly chose TFBSs that have undergone gain or loss events [48] from NRs and NDRs in *S. cerevisiae *promoters for site-directed mutagenesis. Further, we identified the nucleotides that cause TFBS gain or loss in each gene for site-directed mutagenesis. The constructions were performed by PCR-based mutagenesis, which involved two sequential steps [49]. First, the TFBS region of interest in the BY gene was replaced by a *URA3 *cassette with about 45 bp flanking homologous regions to the gene of interest at both ends. To perform the first transformation, we used the LiOAc/SS Carrier DNA/PEG method [50], and the insertion of *URA3 *in the TFBS region was confirmed by diagnostic PCR and sequencing. The inserted *URA3 *was then replaced by a second transformation with the appropriate fragment of BY's PCR-based TFBS-modified sequence (where the specific transcription factor could not bind) in the *URA3*-inserted strain. The second transformation was performed by electroporation based on the user manual of MicroPulser™ electroporator (BIORAD). The transformants were selected by 5-Fluoroorotic Acid (5-FOA) counter selection. Only the strains (called swapped strains) that carried the desired sequence (where the specific transcription factor could not bind) survived and formed colonies on the media with 5-FOA(4 g/ml). The constructions in the TFBS region were confirmed by diagnostic PCR and sequencing.

### Perusing expression shifts with real-time PCR

To compare the mRNA levels of the candidate genes (the genes in the mutagenesis and control groups), we used SYBR green core reaction to perform quantitative PCR (Applied Biosystems model 7,300 Real-Time PCR System). Before performing real-time PCR, total RNAs were first reverse transcribed by a high-capacity cDNA reverse transcription kit (Applied Biosystems) using oligo dT primers as reverse transcription primers. Real-time PCR was performed on the final volume of 25 μL containing 50 ng of the cDNA sample, 50 nM of each gene-specific primer, and 12.5 μL of the SYBER green Taq premixture [51]. The PCR conditions included enzyme activation at 50°C for 2 min and 95°C for 10 min, followed by 40 cycles of denaturation at 95°C for 15 sec, and annealing/extension at 60°C for 1 min. To verify that a single product had been amplified, a dissociation curve was generated at the end of each PCR cycle using the software provided by the Applied Biosystems 7,300 Real-Time PCR System (version 1.4). The relative expression of each gene was normalized to that of the *ACT1 *gene (ΔCt, the Ct (cycle threshold) is defined as the number of cycles required for the fluorescent signal to cross the defined threshold). In addition, the amplification efficiency of each primer pair was tested by using two-fold serial dilutions of the templates as suggested by ABI. Finally, the mRNA levels of the candidate genes were compared using a paired *t*-test.

## Results

### The distribution of TFBSs is constrained by nucleosome occupancy

To understand the differences in the selection constraints due to nucleosome occupancy of the regulatory sequences in yeast promoters, we first compared the distribution of TFBSs in orthologous genes of Saccharomycetaceae and Saccharomyces specific genes in NRs and NDRs. For this task, we downloaded 2,152 genes of *S. cerevisiae *that had orthologs in both *C. glabrata *and *K. lactis *from OrthoMCL-DB [41] with 23,605 TFBSs and 75 Saccharomyces specific genes with 1144 TFBSs (Table 1 see Materials and Methods for details). In Saccharomyces specific genes, frequency of TFBSs was found to be higher in NRs than in NDRs; however, in orthologous genes, TFBSs were more frequent in the NDRs (Table 2). The *p*-value of the two-sided *χ^2^*-test is ≤ 0.05, which indicates a significant association between TFBSs and nucleosome occupancy, rather than random expectation (see Materials and Methods). These results suggest that young genes found only in the descendent *S. cerevisiae *species exhibit more TFBS variation and frequently occur in NRs, indicating a possible source of the vicissitude in their regulatory sequences.

To verify the evolutionary tendency of TFBSs with respect to nucleosome occupancy, we examined the distribution of TFBSs in the promoters of duplicate gene pairs at NRs and NDRs. Our results (one-sided two-sample proportion test; *p*-value < 10^-40^) indicated that the duplicate pairs that have undergone relaxation of the selection constraint [42,52-54] also exhibited more TFBS variation at NRs than at NDRs (Table 2).

### Comparing the evolution rate of TFBSs at NRs and NDRs

**Figure 2:**
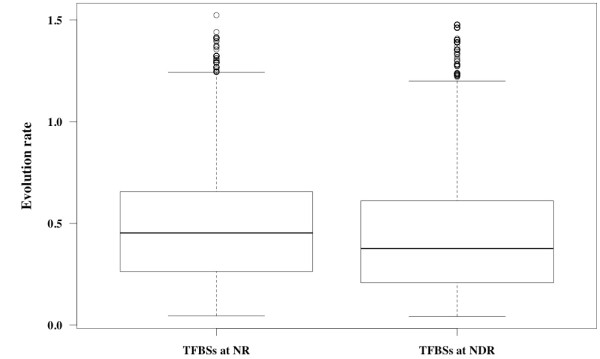
**Evolution rate of TFBSs conserved in *sensu stricto *yeast species at NRs and NDRs using minimum parsimony method**. The evolution rate of TFBSs in the *sensu stricto *species was found to be higher at NRs than at NDRs (Wilcoxon-Mann-Whitney U two-sample test, *p*-value = 1.61×10^-32^).

Previous studies analyzed the dependence of nucleotide substitution rates in the yeast genome by comparing their positions on a map of nucleosome locations [9,25]. A relative difference (about 10%) in substitution rates between the NDR and the equidistant centre point of nucleosomal DNA (dyad) was reported in Washietl *et al. *[25]. In this study, by determining the minimum parsimony of nucleotides at each position (see Materials and Methods), we analyzed the impact of nucleosome occupancy on the evolution rate of TFBSs in *sensu stricto *yeasts (Figure 1g and 1h). We only considered alignments of the sequences available in all five *sensu stricto *species, *i.e*., we excluded regions containing gaps in the alignment. Our dataset contained 21,930 TFBSs. This analysis was performed separately on the TFBSs at NRs and NDRs. Though, our data for evolutionary rate scattered broadly, the median evolution rate of the TFBSs in our dataset (Figure 2), is significantly higher at NRs (0.45) than at NDRs (0.37) according to Wilcoxon-Mann-Whitney U two-sample test (*p*-value = 1.61×10^-32^). Nevertheless, experimental errors in determining nucleosome positions and TFBS prediction might be the possible source of the broad scatter in the data and could bias our result.

### TFBS gain and loss events in NDRs show higher possibility of altering gene expression

To evaluate the impact of TFBS change at NR and NDR on gene expression, we randomly selected six TFBSs that were known to have experienced gain or loss events [48] from NDRs and NRs in *S. cerevisiae *promoters for site-directed mutagenesis. The TFBSs corresponding to gain or loss events were removed from the laboratory strain (Additional file 2 Table S2). After which, we measured the expression changes in mutant/wild type strains using quantitative PCR (real-time PCR). In the six mutagenesis cases in both NRs and NDRs (*t*-test *p*-value < 0.05), significant expression changes were found between the mutant and wild type strains in three TFBSs in NDRs (50%), while only one out of six TFBSs in NRs demonstrated expression differences (Table 3). These results indicate that TFBS gain or loss events at NDRs may have a higher probability of causing expression differences than those at NRs.

**Table 3:** Expression changes in genes with swapped mutants and wild type.

		SWAP		BY(WT)		
**Systemic name**	**TFBS**	**ΔCt Target/Act1**	**STDEV**	**ΔCt Target/Act1**	**STDEV**	**Nucleosome** **occupancy**

**YBR248C**	**ABF1**	3.091	0.167	2.443	0.070	NDR*
**YDR519W**	**HAC1**	3.648	0.012	2.846	0.050	NDR
**YGL253W**	**RGT1**	-0.259	0.179	-0.192	0.026	NDR
**YLR214W**	**RCS1**	3.245	0.270	2.747	0.079	NDR*
**YLR450W**	**ROX1**	1.757	0.236	1.688	0.031	NDR
**YPL111W**	**ABF1**	0.245	0.013	0.075	0.056	NDR*
**YAL054C**	**CAT8**	6.233	0.225	6.145	0.014	NR
**YDR072C**	**PDR3**	5.574	0.024	5.363	0.001	NR
**YGL255W**	**ZAP1**	7.008	0.048	5.435	0.004	NR*
**YKL175W**	**ZAP1**	2.685	0.184	2.788	0.013	NR
**YML075C**	**ROX1**	1.871	0.087	1.732	0.099	NR
**YNL117W**	**CAT8**	-1.444	0.056	-1.331	0.126	NR

Twelve genes that experienced TFBS gain or loss events at both NDRs and NRs were selected for site-directed mutagenesis and expression analysis. T-test was based on ΔCt of swapped mutants and BY4741(wild type).

* Significantly different from ΔCt^BY^(*p*-value< 0.05) by *t*-test

## Discussion

Evolutionary analysis in promoter regions has provided important insights into the regulatory process and the properties of TFBS motifs [30-32,48]. Yet, the current understanding of TFBS evolution is limited, especially in deciphering the extant of the contributions from other DNA-binding factors such as nucleosomes, chromatin remodelers and chromatin modifiers. While there are several theories highlighting the influence of chromatin architecture, specifically the nucleosome landscape on the molecular evolution of genomic DNA [9,25], not many studies focus on the role played by nucleosome in TFBS evolution [34,55,56]. Further, studies have partially resolved the effect of nucleosome arrangement patterns on transcription [18,57]. Our goal here is to comprehensively elucidate the evolution of TFBSs due to the constraints on sequence structure affected by nucleosome positioning in *sensu stricto *yeasts. We have conducted a detailed evolutionary analysis of TFBSs with respect to nucleosome occupancy by taking advantage of recently published nucleosome map in *S. cerevisiae *[11]. Our analysis has uncovered TFBS evolution changes in the context of nucleosome occupancy by different perspectives.

Our results suggest that the evolution of TFBSs in yeast species has a noteworthy relationship with the nucleosome organization encoded in promoter sequences on a genome-wide scale. We found that TFBSs in orthologous genes (shared in Saccharomycetaceae) were frequently located at NDRs, while TFBSs in younger Saccharomyces specific genes were dominant at NRs. Furthermore, genes that have undergone duplication are known to be under lower purifying (stabilizing) selection [54,58]. In addition, promoters near duplicate gene pairs are also known to have increased substitution rates, indicating relaxation of selection constraints [53]. According to our results, the TFBSs at NRs in duplicated genes exhibited more variation in terms of their occurrence frequency than those at NDRs. Consistently, the expression divergence of duplicate genes confirms rapid evolution, which could be attributed to *cis-*changes, specifically to the variation of TFBSs [42,59]. These results are also concordant with our findings for TFBSs in ancestral and young gene sets, reinforcing the possibility of difference in selection across NRs and NDRs. A possible source of difference could be ascribed to the impinging of repair mechanisms of DNA sequences by nucleosomes [60-63]. This is reflected by, high mutation rates at NRs than at linker regions, which are depleted of nucleosomes [9,25] and could conceivably explain the frequent occurrence of novel TFBSs in these regions. In addition, a recent study has suggested that natural selection acts to maintain genome-wide signature of nucleosome formation [64]. This study also provided evidence for selection on conserving chromatin structure, and contributes significantly in driving mutational bias at both coding and non-coding regions. Most importantly, the above results reveal the significance of conglomerate analysis of regulation and promoter nucleosome status in explaining the regulatory evolution [55].

The availability of whole genome nucleosome maps has facilitated research on the regulatory process. As a result, some studies have hinted that the existence of competition and co-operation between nucleosomes and transcription factors may contribute to the regulatory effects on expression divergence [26,65-67]. Since regulatory sequences are believed to play an important role in molecular evolution [48,68,69], we explored the evolutionary significance of the dominance of TFBSs in young genes located at NRs by comparing the evolution rates of TFBSs at NRs and NDRs. Our results demonstrated that, at NRs, TFBS evolutionary rates were significantly higher than at NDRs, although the data seems to be broadly scattered. This indicates the possibility that NRs, which can accommodate more TFBSs variations, may contain binding site sequences with lower purifying selection relative to NDRs. The finding is also congruent with the recent work of Babbitt [70], which indicated that the nonfunctional TFBS could escape purifying selection when they occur in high nucleosome occupancy. It is likely that the weaker selection constraint on TFBSs at NRs plays an important role in the creation of novel binding sites *via *stochastic mutational processes [36,71]. Furthermore, the weaker selection constraint at NRs can probably be explained by the fact that DNA in nucleosomes is less accessible to DNA binding proteins [72].

Functional constraint could be one of the major explanations for the different evolution rates in NRs and NDRs. Therefore, it is crucial to investigate whether there is a difference in impact of TFBS changes on expression at NRs and NDRs. We provided an indirect evidence *via *TFBS modification and expression analysis (Table 3 and Additional file 2 Table S2) and revealed that a larger fraction of swapped mutants at NDRs led to expression shift than swapped mutants at NRs. Although our data is limited, previous studies in several species, including yeast have also indicated the role played by nucleosome in regulating gene expression [18,26,57]. These results suggest the possibility of difference in selection constraint on TFBSs at NRs and NDRs.

## Conclusions

Recent studies have indicated that nucleosome organization broadly influences regulatory evolution in yeast [27,55]. For example, in the evolution of within species *cis*-regulatory elements, it is known that polymorphism in the regulatory sequences are interrelated to changes in nucleosome occupancy [73,74]. The data from our current analysis shows that NRs can contain more TFBS variations, which in turn reflects the importance of TFBSs located in NDRs [75]. We confirmed the difference in selection constraint at NRs and NDRs by measuring the evolutionary rates of TFBSs at these regions Moreover, observations reported in literature support our findings by demonstrating the differences in the accessibility of DNA to their binding proteins inside and outside nucleosome occupied regions [60,62,72]. To ensure the quality of our data, we took several precautions in data selection and have controlled for possible source of bias in our estimates. Thus, the current analysis of the effect of nucleosome positions on the evolution of TFBSs can be considered reliable. Though our study reveals an important feature in TFBS regulatory evolution, a more direct analysis would be required to address the nature of selection that drives the distinction in evolutionary rates.

## Authors' contributions

KBSS, HKT and DW designed the analysis. KBSS and WYC performed analysis. CYW performed site-directed mutagenesis experiment. KBSS, HKT and DW wrote the paper and analyzed the data. HKT and DW were the principal investigators and conceived the experimental design and analysis. All authors read and approved the final manuscript.

## Supplementary Material

Additional file 1 **Table S1**. The list of orthologs and *S. cerevisiae *specific genes used in this study. Click here for file

Additional file 2 **Table S2**. The details of TFBSs (that had undergone gain or loss events) used in the site-directed mutagenesis experiment along with their promoter and target gene information. Click here for file
