# Supplementary material for: Evidence of association between Nucleosome Occupancy and the Evolution of Transcription Factor Binding Sites in Yeast
Source: BMC Evol Biol. 2011 May 31;11:150. doi: 10.1186/1471-2148-11-150 (PMC3124427; doi:10.1186/1471-2148-11-150)
Supplement: Additional file 2 — Table S2. The details of TFBSs (that had undergone gain or loss events) used in the site-directed mutagenesis experiment along with their promoter and target gene information. [file 1471-2148-11-150-S2.PDF]

Supplementary Table S2: Details of TFBSs that have undergone gain/loss events used in the site directed mutagenesis experiment along with their promoter and target gene information

TF	Target Gene	TFBS state					TFBS information in Scer			
		S. cer	*S. bay*	*S. kud*	*S. mik*	*S. par*	Nucleosome state	TFBS	Start	End
**ABF1**	**YBR248C**	+	-	+	+	+	**NDR**	TCTCTCTCCACG	217	228
**HAC1**	**YDR519W**	+	+	-	+	+	**NDR**	CCAGC	150	154
**RGT1**	**YGL253W**	+	+	+	-	+	**NDR**	TTTTCCG	329	335
**RCS1**	**YLR214W**	+	+	-	-	+	**NDR**	TACACCCA	421	428
**ROX1**	**YLR450W**	+	+	-	+	+	**NDR**	CGCATTGTT	333	341
**ABF1**	**YPL111W**	+	+	+	-	-	**NDR**	CGTCGCCCGCGAT	652	664
**CAT8**	**YAL054C**	+	-	+	-	+	**NR**	TCCATTTCGCCGG	504	516
**PDR3**	**YDR072C**	+	-	+	+	+	**NR**	CTTTCCGCGGAA	544	555
**ZAP1**	**YGL255W**	+	-	-	+	+	**NR**	ACCCTCAAGGT	681	691
**ZAP1**	**YKL175W**	+	-	-	-	+	**NR**	ACCTTAAGGGT	651	661
**ROX1**	**YML075C**	+	+	-	-	+	**NR**	CGGGTGATGG	437	446
**CAT8**	**YNL117W**	+	+	+	-	+	**NR**	TCCATTGGGCCGA	292	304
